# A new score statistic to test for association given linkage in affected sibling pair-control designs

**DOI:** 10.1186/1753-6561-1-s1-s39

**Published:** 2007-12-18

**Authors:** Jeanine J Houwing-Duistermaat, Hae Won Uh, Hans C van Houwelingen

**Affiliations:** 1 Department of Medical Statistics and Bioinformatics, Leiden University Medical Center, Postzone S-5-P, PO Box 9600, 2300 RC, Leiden, The Netherlands

## Abstract

To detect association of the DR1 allele with rheumatoid arthritis (RA) given linkage in the affected sibling pairs of the replicates of Problem 3 of Genetic Analysis Workshop 15 (GAW15), we propose a new score statistic that takes into account the linkage information. We knew the answers. Linkage studies are often followed by case-control association studies of candidate genes located under the peak to identify the causes of a linkage peak. One strategy is to type the affected sibling pairs from the original linkage study and a set of unrelated controls for single-nuclear polymorphisms describing the genetic variation of these genes. For this affected sibling pair-control design, we propose a relative-risk model for the relationship between the disease outcomes of sibling pairs and their genotypes and identity-by-descent status at the locus of interest. From this model, we derive a score statistic to analyze genetic association given linkage. We compare the performance of the new statistic to the method of Li et al. and to a standard association analysis that neglects the information on the identity-by-descent status of the sibling pair. We conclude that for the GAW15 data the new method performs well and that methods that use the linkage information may be more efficient than standard comparisons of genotypes in cases and controls.

## Background

Genome-wide linkage analyses are often followed by association studies of candidate genes located under the linkage peak using a case-control design. With these genetic association studies one hopes to identify candidate genes whose variation causes the excess identical-by-descent (IBD) sharing of marker alleles in the linkage study. Single-nucleotide polymorphisms (SNPs) describing the genetic variation of the studied genes are typed in a set of controls and a set of cases and the genotype distributions are compared between cases and controls. Often a part of or all cases originates from the linkage study. Li et al. [1] studied the efficiency of various designs for genetic association studies. In this paper we consider the affected sibling pair (ASP) – control design. Here, the controls are unrelated healthy subjects. We use the available linkage information in the ASPs to obtain an efficient statistic to test the null hypothesis of no association given linkage versus the alternative of association.

We first derive a relative-risk model for the disease outcomes with a term modeling association between the candidate SNP and the outcome and a term modeling excess IBD sharing at the SNP locus. This is a model for joint linkage and association. From the likelihood of this model, we derive a score statistic for testing association given linkage. The new score statistic appears to be a stratified analysis of the genotype distributions according to the IBD status of the sibling pair at the SNP locus. If the SNP is one of the causes of excess IBD sharing in the region, the stratified analysis should be more efficient than an unstratified analysis, which neglects the IBD information. The unstratified analysis compares the genotype frequencies of the affected sibling pairs and the controls.

Recently, a model linking disease outcomes in sibling pairs to linkage and association information measured at a candidate SNP was proposed by Li et al. [2]. The relationship between disease outcomes and the candidate SNP is modeled in terms of penetrances of the disease genotypes and disease-SNP haplotype frequencies. This model with six parameters is the most general model (GM). The model for linkage only (LE) has three parameters, namely two for the IBD probabilities at the SNP location and one for the allele frequency of the candidate SNP. The difference between the log of the likelihood of the GM model and the log of the likelihood of the LE model forms a test for association given linkage. The program LAMP can be used to compute the likelihood ratio test statistics and corresponding *p*-values. A disadvantage of the method is that it models one single gene under the peak, which may not be realistic for complex genetic traits studied in sibling pairs who share a relatively large region. Further likelihood ratio tests may be more sensitive for model deviations than score statistics [3].

We apply the new score statistic, the unstratified comparison of genotype frequencies between the affected sibling pairs and controls, a statistic which combines the new score statistic and the unstratified statistic and the likelihood ratio method of Li et al. [2] to the ASP and controls of the 100 replicates of Problem 3. The DR locus is used as the candidate locus. At the DR locus, two variants exist which increase the risk for disease, namely DR1, with a modest effect, and DR4, with a large effect. Because the effect of DR4 is large and we are interested in detecting small effects, we removed siblings and controls homozygous for the DR4 allele. Our goal was to identify the small effect of the DR1 allele. In these data the marker information is almost perfect. Therefore, we derived the score statistic under the assumption of known IBD. In the discussion we describe how to adapt the statistic when uncertainty in IBD status exists.

## Methods

### New score statistic

Let *G *measure the total effect of the unobserved genes under the identified linkage peak. Without loss of generality, we assume that *G *has mean value of zero. Further, we assume that the disease is rare. The relative effect RR of *G *is modelled by RR = exp(*G*). By using second-order Taylor approximations around *G *= 0, the conditional probability of being affected given *G *is proportional to (1 + *G *+ 0.5*G*^2^) and the probability that two siblings are affected given their genetic effects *G*_1 _and *G*_2 _is proportional to (1 + *G*_1 _+ *G*_2 _+ 0.5 *G*_1_^2 ^+ 0.5 *G*_2_^2 ^+ *G*_1_*G*_2_).

The information available on *G *is for each sibling pair, the pair of genotypes at the candidate SNP of interest (S_1_, S_2_) and the IBD status at the candidate locus IBD_S_. The conditional probability that two siblings are affected given (S_1_, S_2_) and IBD_S _is proportional to (1 + E(*G*_1_|S_1_) + E(*G*_2_|S_2_) + 0.5 Var(*G*_1 _+ *G*_2_|S_1_, S_2_, IBD_S_)). By applying Bayes rule and assuming Var(*G*_1 _+ *G*_2_|S_1_, S_2_, IBD_S_) = Var(*G*_1 _+ *G*_2_|IBD_S_), we obtain

$$\begin{array}{lll} {\text{P(S}}_{\text{1}}{\text{,S}}_{\text{2}}{\text{,IBD}}_{\text{S}}\text{|ASP)} & \approx & \frac{{\text{P(S}}_{\text{1}}{\text{,S}}_{\text{2}}{\text{,IBD}}_{\text{S}}\text{)}}{\text{P(ASP)}}\text{(1}+{\text{E(G}}_{\text{1}}{\text{|S}}_{\text{1}}\text{)}+{\text{E(G}}_{\text{2}}{\text{|S}}_{\text{2}}\text{)}+1/2{\text{Var(G}}_{\text{1}}+{\text{G}}_{\text{2}}{\text{|IBD}}_{\text{S}}\text{))}\\ & \propto & \frac{{\text{P(S}}_{\text{1}}{\text{,S}}_{\text{2}}{\text{,IBD}}_{\text{S}}\text{)}}{\text{P(ASP)}}\text{(1}+\beta {\text{(S}}_{\text{1}}+{\text{S}}_{\text{2}}-\text{2}\mu \text{)}+\delta {\text{(IBD}}_{\text{S}}-\text{1)/}\sqrt{\text{2}}\text{),} \end{array}$$

with *μ *equal to E(S_1_). By using Var(*G*_1 _+ *G*_2_|S_1_, S_2_, IBD_S_) = Var(*G*_1 _+ *G*_2_|IBD_S_), we assume that given the IBD status, the variance and covariance of the genetic effects do not depend on the SNP genotypes. The parameter *δ *measures linkage at the SNP location and the parameter *β *measures association of the SNP to the disease. The model extends the model of Kong and Cox [4] by also including an association term.

Now the log likelihood function is given by

$$\ell (S_{1},S_{2},IBD_{S}|ASP)=c+\sum_{sib\;pairs}\ln(1+\beta (S_{1}+S_{2}-2\mu )+\delta (IBD_{S}-1)/\sqrt{2}),$$

where *c *is a constant independent of *β*. The corresponding score statistic U to test the null hypothesis H_0_: *β *= 0 given IBD_S _is given by

$$U=\sum_{IBD=0}\frac{1}{1-\delta }(S_{1}+S_{2}-2\mu )+\sum_{IBD=1}\left(S_{1}+S_{2}-2\mu \right)+\sum_{IBD=2}\frac{1}{1+\delta }(S_{1}+S_{2}-2\mu ).$$

The parameter *δ *can be obtained by applying Kong and Cox method [4] to the ASP, and the parameter *μ *can be obtained from the controls. Under the null hypothesis the statistic U has mean value of zero. The variance of U can be empirically estimated or computed based on the genotype frequencies under the null hypothesis.

The unstratified version of this statistic tests the null hypothesis of no association without accounting for linkage information. Also, for this statistic the genotypes of sibling pairs are not independent. The variance of the statistic can be empirically estimated. If the assumption of Var(*G*_1 _+ *G*_2_|S_1_, S_2_, IBD_S_) = Var(*G*_1 _+ *G*_2_|IBD_S_) is violated the stratification according to the IBD groups will not be optimal. The test statistic is still valid, but the gain in power compared to the unstratified test statistic will be smaller. Therefore, we propose also the statistic U*, which combines the unstratified statistic and the new score statistic by pooling the one and two IBD groups.

### Materials

To evaluate the performance of the new statistics U and U*, we analyzed the sibling pairs affected with RA of Replicates 1 to 100. For the simulation of the replicates, a lifetime prevalence of RA of 0.0107 and a lambda-sib (lifetime prevalence for siblings of affected individuals divided by the population prevalence) of 9.03 was used. The marker information was high due to a dense map (average spacing of 5 cM), a high marker heterozygositiy (above 0.7), and the availability of parental genotypes. For association, we studied the DR locus with two risk variants (DR1 and DR4). The DR1 allele has a frequency of 0.1 and a genotype relative risk for homozygous carriers versus homozygous carriers of the DRx allele of 1.5. The other variant DR4 has a frequency of 0.25 and increases the risk for RA enormously. The genotype relative risk for homozygous carriers DR4 versus homozygous carriers of the DRx allele is 30. Our aim was to identify the DR1 allele at the DR locus.

We first removed the sibling pairs and controls who are homozygous carriers of the DR4 allele. The number of affected sibling pairs used for analysis varied from 211 to 270 with a mean number of 238 sibling pairs. The number of homozygous carriers in the controls was small, and around 2000 controls were available for association analysis. For each replicate we used Merlin [5] to estimate the parameter *δ *and to compute the multipoint IBD_S _at the DR locus, assuming an additive model for each sibling pair. For almost all sibling pairs, the IBD status was observed. When the IBD status was uncertain, the most likely IBD status was assigned to the sibling pair. In the 100 replicates, the parameter *δ *varied from 0.13 to 0.36 with a mean of 0.25. The genotype *S*_*k *_for *k *= 1 or 2 was defined as the number of DR1 alleles carried by sibling *k *and its expectation *μ *was computed from the controls. In the replicates, the parameter *μ *varied from 0.28 to 0.37 with a mean of 0.32.

We applied the new score statistic U, the unstratified test statistic and the statistic U*, which combines the sibling pairs who share two alleles IBD and one allele IBD. We estimated the variances of the three statistics empirically. Finally, for the method of Li et al. [2] we used the program LAMP assuming an additive model and a disease prevalence of 0.01. We considered both the ASP design as well as the ASP-control design. Note that we did not include the uncertainty of the parameter *μ *in the computation of the *p*-value for the score statistics. To be able to compare the performance of the score statistics with the performance of the Li method, we made the uncertainty in the estimated allele frequencies in the controls negligible by multiplying each control record four times.

## Results

In Table 1, the *p*-values of the various statistics applied to the first five replicates are given. Table 2 shows the number of times the null hypothesis of no association is rejected in the 100 replicates using the various methods. From comparing the results of LAMP using the ASP-control design with the ASP design it is clear that including the controls increases the power to detect association. At the 5% level all methods using the ASP-control design were able to reject the null hypothesis of no association in all replicates. At the 0.1% significance level, the statistic U* and the LAMP method perform best, but the differences with the original statistic U and the unstratified statistic are small. Note also that the unstratified statistic performs a little better than the stratified U statistic.

**Table 1:** *p*-Values for various methods used for testing for genetic association

Replicate	Number of pairs	*μ*^a^	*δ*^b^	*P*-values for testing association in ASP-controls^c^	*P*-values of various statistics for testing association given linkage			
					
					U^d ^in ASP-controls	U*^e ^in ASP-controls	LR statistic^f ^in ASP-controls	LR statistic^f ^in ASP
1	229	0.31	0.34	5.6 × 10^-5^	1.8 × 10^-5^	2.2 × 10^-8^	8.0 × 10^-5^	0.4
2	225	0.34	0.24	1.4 × 10^-6^	3.1 × 10^-6^	1.2 × 10^-9^	7.0 × 10^-5^	0.8
3	223	0.32	0.31	1.1 × 10^-4^	1.4 × 10^-4^	1.0 × 10^-7^	7.1 × 10^-4^	0.6
4	214	0.33	0.27	8.8 × 10^-4^	1.2 × 10^-3^	1.8 × 10^-5^	0.009	0.6
5	244	0.35	0.23	1.0 × 10^-3^	1.7 × 10^-3^	5.6 × 10^-5^	0.008	0.2

^a^Expectation of number of DR1 alleles carried by an individual

^b^Kong and Cox *δ *[4]

^c^The dependency of the genotypes of siblings is taken into account by using the empirical variance

^d^New score statistic

^e^Modified version of U obtained by combining siblings with IBD = 1 and IBD = 2

^f^Likelihood ratio statistic of LAMP comparing the models GM and LE [2]

**Table 2:** Number of times H_0 _is rejected using the various methods of testing for genetic association

			Testing for association given linkage			
			
Replicates	Significance level	Testing association in ASP-controls^a^	U^b ^in ASP-controls	U*^c ^in ASP-controls	LR statistic^d ^in ASP-controls	LR statistic^d ^in ASP
1 to 100	0.05	100	100	100	100	42
1 to 100	0.001	93	91	97	98	5

^a^The dependency of the genotypes of siblings is taken into account by using the empirical variance

^b^New score statistic

^c^Modified version of U obtained by combining siblings with IBD = 1 and IBD = 2

^d^Likelihood ratio statistic of LAMP comparing the models GM and LE [2]

## Discussion

The new statistic U* performs as well as the likelihood-ratio statistic of Li et al [2]. The method of Li et al. performs well with these data although the assumption of one single gene under the linkage peak is violated (DR1 allele, DR4 allele, locus C, and locus D). The score statistic U does not assume one gene under the peak, but it assumes that Var(*G*_1 _+ *G*_2_|S_1_, S_2_, IBD_S_) = Var(*G*_1 _+ *G*_2_|IBD_S_). This assumption does not hold when multiple disease variants or disease loci are in linkage disequilibrium (LD) with the candidate SNP. Thus the statistic U allows for multiple disease loci as long as they are not in LD with the candidate SNP. When this assumption is violated, the stratification is not optimal and the statistic loses power. An ad hoc solution is the modified statistic U*, which combines the unstratified statistic and the new statistic. Probably due to the presence of the DR4 allele in the reference category and the LD between the DR locus and locus C, U* and the unstratified statistic perform better than the score statistic U. The method of Li et al. models a disease locus in LD with the candidate SNP and is therefore more flexible to deal with multiple disease variants. Extended simulations are needed to study the performance of these statistics under multiple disease loci models.

When multiple variants exist or multiple disease loci are present in the LD block around the candidate SNP, the statistic U is still valid, but loses power. In this paper, we used the ad hoc U* to deal with the presence of the DR4 allele in the reference category. More sophisticated solutions are warranted. When multiple variants are observed, the model could be extended by including more *S*_*k *_variables in the model corresponding to the different risk alleles. When these other variants are unknown, the parameter of the linkage term should depend on the genotypes *S*_*k*_, for example by inclusion of interaction terms between the association and linkage information in the model.

The version of the score statistic presented in this paper assumes observed IBD for the sibling pairs. The statistic can easily be adapted to deal with uncertainty in IBD status by using a weighted statistic with weights equal to the IBD probabilities. Another extension is to allow for haplotype ambiguity. However this is not straightforward because the haplotypes of sibling pairs are not independent. The control population is rather large in the GAW15 data, we assumed known population frequencies of the DR1 genotypes. For smaller populations the uncertainty in the estimates can be taken into account by computing the variances of the statistic under the null hypothesis.

Similar to the models of Li et al. [2], the relative-risk model that is presented in this paper can also be used for joint linkage and association analysis and for analysis of residual linkage given association. To analyze joint linkage and association in the GAW14 data sets, we [6] used the conditional logistic models of Olson [7]. This method models the relationship between the IBD probabilities in affected relative pairs and covariates. The SNP genotypes can be included as a covariate in the model. The MASC method [8] also considers the IBD status and observed genotypes. The method classifies index patients into categories based on marker genotypes and IBD status and tests for deviations of the distribution within these classes using chi-square statistics. Finally, the method of Sun et al. [9] tests the null hypothesis of no residual linkage given association. The method stratifies sibling pairs according to the siblings' genotypes at the SNP locus and compares the IBD distribution in the strata to its expectation. The derivation of the statistics from our model and comparisons with existing methods are topics for future research.

## Conclusion

We conclude that the relative-risk model provides a new framework for joint linkage and association analysis. When testing the null hypothesis of no association, more efficient statistics may be obtained when the linkage information is used. To test the null hypothesis of no association given linkage, the modified score statistic U* performs as well as the method of Li et al. in detecting the effect of the DR1 allele in the replicates of Problem 3.

## Competing interests

The author(s) declare that they have no competing interests.
